# Erythro-Melalgia

**Published:** 1891-03-28

**Authors:** 


### The Practitioner's Mirror and Retrospect.

[*The Editor will be glad to receive offers of co-operation and contributions from members of the profession. All letters should be addressed to* The Editor, The Lodge, Porchester Square, London, W.]

### MEDICINE.

### ERYTHRO-MELALGIA.

This is the term applied by Dr. Weir-Mitchell to an affection of the extremities. It is a vaso-motor neurosis, and Dr. Weir-Mitchell is of opinion that the vaso-motor phenomena are caused either by a trupain, as a reflex condition, or by an implication of the vaso-motor centres, or by disease of the central organs, the disease then giving rise to the pain. The characteristic symptoms usually begin with pain in one or both feet, most frequently in the ball of the great toe or of the foot, or in the heel. Sometimes the pain extends to the dorsum of the foot, and even very rarely to the leg. Most frequently it is confined to one or both soles. The pain is burning in character, and as the disease advances is often very severe, or even agonizing during the paroxysms. In the earlier stages it is usually associated with prolonged standing or excessive walking; later, it comes on whenever the patient assumes the upright posture, or when the feet hang down. It is relieved by the horizontal position and by cold, while it is much aggravated by warmth, even the heat of the bed-clothes becoming intolerable. The feet are red, or, in severe cases, assume a dusky-red colour whenever the upright position is assumed. It is a rare disease, and therefore the case reported by Dr. A. V. Wendel, in the New York *Medical Journal* for November 15th last, will be interesting. His patient, a female, aged sixty-two, had borne four children, and was by occupation a nurse. Her parentage had been healthy. Her own health had always been good up to the previous April. At that time she was nursing a case of puerperal septicæmia, and a number of times daily had to wring out cloths dipped in hot carbolic solution. After doing this for several days she noticed her feet to be very painful, and, on examination, blue and swollen on the anterior portion. These symptoms became so severe as to oblige the patient to give up her profession and take to bed. The pain was excruciating, especially at night and on pressure, the attacks coming on paroxysmally. When she came to Dr. Wendel she was much emaciated, had atheromatous arteries, obstinate constipation, the feet presenting over the anterior and lateral aspects, especially on the plantar surfaces, irregular bluish-purple spots, very sensitive, and sweating freely. Urinalysis gave negative results. The treatment consisted of a ferruginous tonic, the usual hygienic measures, and cold effusions locally; but while the general condition improved, the local did not. The local application of belladonna, hot water, &c., failed. Electricity only aggravated it. Dr. Wendel then decided to put her on arsenic, which was pushed till the physiological symptoms became manifest. After several days of this treatment the patient began to improve, and six days after walked around the block with ordinary shoes, being the first time in many months that she had been able to tolerate any covering on her feet. Improvement went on so rapidly, and the arsenic was stopped without recurrence of the symptoms. Dr. John Morton has given a detailed account of the diseases, and reported cases. In one very intractable case, in which electricity, massage, and various other local and general remedies had failed to give relief, an almost complete recovery was obtained by the subcutaneous injection of morphia and atropia, tried as a last resource. The needle of the syringe was passed directly into the sole of the foot on the inner side. This procedure was practised twice daily for about three weeks, beneficial effects manifesting themselves almost at once.

